# Characteristics of subsequent contralateral proximal femoral fracture: more convenient access is needed to treat osteoporosis

**DOI:** 10.1186/s13018-023-03621-y

**Published:** 2023-02-21

**Authors:** Yuxuan Jiang, Yangjun Zhu, Binfei Zhang, Dongxu Feng

**Affiliations:** 1 Department of Orthopaedic Trauma, Hong Hui Hospital, Xi’an Jiaotong University School of Medicine, Xi’an, 710054 Shaanxi Province China; 2 Department of Joint Surgery, Hong Hui Hospital, Xi’an Jiaotong University School of Medicine, Xi’an, 710054 Shaanxi Province China

**Keywords:** Hip fracture, Fragility fracture, Proximal femoral fracture, Subsequent femoral fracture, Osteoporosis treatment

## Abstract

**Background:**

Patients with proximal femoral fracture (PFF) have high mortality and many complications. Osteoporosis increases the risk of subsequent fractures, leading to subsequent contralateral PFF. This study was performed to analyze the features of individuals with subsequent PFF following surgical therapy of first PFF and to ascertain whether such patients received an examination or treatment of osteoporosis. The reasons for lack of examination or treatment were also analyzed.

**Methods:**

This retrospective study involved 181 patients with subsequent contralateral PFF who underwent surgical treatment in Xi'an Honghui hospital from September 2012 to October 2021. The patients’ sex, age, hospital day, mechanism of injury, surgical procedure, fracture interval, fracture type, fracture classification, and Singh index of the contralateral hip at the time of the initial and subsequent fractures were recorded. Whether the patients took calcium and vitamin D supplements, used anti-osteoporosis medication, or underwent a dual X-ray absorptiometry (DXA) scan was recorded, as was the start time of each. Patients who had never undergone a DXA scan or received anti-osteoporosis medication took part in a questionnaire.

**Results:**

The 181 patients in this study comprised 60 (33.1%) men and 121 (66.9%) women. Patients with initial PFF and subsequent contralateral PFF had a median age of 80 years (range 49–96 years) and 82 years (range 52–96 years), respectively. The median fracture interval was 24 (7–36) months. Contralateral fractures occurred at the highest incidence between 3 months and 1 year (28.7%). The Singh index was not significantly different between the two fractures. In 130 (71.8%) patients, the fracture type was the same. No significant difference was found in the fracture type or fracture stability classification. A total of 144 (79.6%) patients had never received a DXA scan or anti-osteoporosis medication. The main reason for not treating osteoporosis further was concern about the safety of drug interactions (67.4%).

**Conclusions:**

Patients with subsequent contralateral PFF were of advanced age, had a higher proportion of intertrochanteric femoral fractures, had more severe osteoporosis, and had longer hospital stays. The difficulty managing such patients requires multidisciplinary involvement. Most of these patients were not screened or formally treated for osteoporosis. Advanced-age patients with osteoporosis need reasonable treatment and management.

## Background

Osteoporosis is a chronic disease that is asymptomatic and “silent.” Often, patients do not pay attention to bone mass reduction until a fracture occurs. This characteristic makes asymptomatic people less likely to aggressively seek medical attention. According to the International Osteoporosis Foundation, one in three women and one in five men aged > 50 years may develop an osteoporotic fracture in their lifetime [1]. Vertebral, wrist, and hip fractures are traditionally recognized major fragility fractures. Hip fracture mainly refers to proximal femoral fracture (PFF), which constitutes 14% of all fragility fractures and accounts for 72% of the total cost of all types of fractures [2].

Young people or people with normal bone mineral density (BMD) generally only sustain hip bone contusion after low-energy injury, and PFF does not readily occur. However, low-energy injury in patients with severe osteoporosis can lead to PFF. Research on high-energy unstable PFF has increased in recent years, but the vast majority of PFFs occur in advanced-age patients with osteoporosis; additionally, more than 80% of people aged > 75 years who develop PFF have sustained such fractures by low-energy injury mechanisms [3]. Osteoporosis greatly increases the risk of fracture, and a prior fragility fracture increases the risk of a second fracture. The incidence of a second major osteoporotic fracture within 1 year, 2 years, and 5 years after a first hip fracture is 1.7%, 4.0%, and 9.0%, respectively. The mortality rate associated with hip fracture 1 to 5 years postoperatively is the highest among all types of fracture [4].

Upon detecting the first low-energy fracture in a patient, physicians should immediately begin diagnostic and treatment protocols for osteoporosis because fracture is the primordial clinical manifestation of osteoporosis. Research shows that the rate of treatment with anti-osteoporosis medication after surgical intervention for the first fragility fracture is higher now than 10 years ago [5]. In various countries, however, there is a large gap between the number of patients who need osteoporosis treatment and the number of patients who actually receive osteoporosis treatment [6, 7].

The incidence of osteoporotic fractures in the Chinese mainland is steadily increasing [8]. Although the awareness of osteoporosis prevention has improved in China in recent years, many patients with osteoporosis still have not received examination and treatment. The incidence of hip fragility fracture remains high. The purpose of this study is to enable orthopedic surgeons to focus on the diagnosis and treatment of osteoporosis in patients with PFF and to understand the reasons for the lack of osteoporosis diagnosis and treatment in this patient population.

## Patients and methods

The study was approved by the Ethics Committee of Hong Hui Hospital (No. 202301014). The authors conducted this research in line with the ethical responsibilities established by the World Medical Association and the Declaration of Helsinki. Written informed consent was not required because this study involved only a review of clinical records.

### Study population

We retrospectively examined the data of adult patients who were diagnosed with subsequent contralateral PFF and hospitalized from September 2012 to October 2021 in Xi'an Honghui hospital and who had received surgical management of a first PFF. Patients with suspected pathological fractures, periprosthetic fractures, or incomplete medical records and patients without preoperative X-ray films of the initial and subsequent contralateral PFFs were excluded.

Each patient’s sex, age, hospital day, mechanism of injury, surgical procedure, fracture interval, fracture type, fracture classification, and Singh index of the contralateral healthy proximal femur at the time of the initial fracture and the contralateral fractured proximal femur were recorded.

According to the data in the medical records, the injury mechanism was categorized as a fall (standing or sitting height or lower), traffic accident, or fall from a height (higher than 2 m). The main fracture types were femoral neck fracture, intertrochanteric fracture, and subtrochanteric fracture. The fracture imaging data were classified by three orthopedic surgeons according to the 2018 new AO classification. Trained surgeons participated in the film reading. If two or more results were obtained, three doctors discussed the finding to determine the final classification. The Singh index was assessed in the same way. The Singh index is mainly used to evaluate the distribution of proximal femoral trabeculae on a hip X-ray image and is classified from grade I to VI. Grade I represents severe osteoporosis, while grade VI represents good trabecular bone density [9]. The classifications of femoral neck fracture and intertrochanteric fracture were dichotomized to determine whether the fracture patterns were stable or unstable. According to the new AO classification, the stable patterns of femoral neck fracture were B1.1, B1.2, B2.1, and B3; all other classifications were considered unstable [10–13]. The stable patterns of intertrochanteric fracture were A1.2 and A1.3; all other fractures were considered unstable [14].

### Follow-up contents

The follow-up data included the treatment or examination status of osteoporosis and the all-cause mortality rate. The distributions of the start times of calcium and vitamin D supplementation, anti-osteoporosis medications, and dual X-ray absorptiometry (DXA) scanning relative to the initial and subsequent contralateral PFF were recorded through telephone or outpatient service. Anti-osteoporosis medications approved by the U.S. Food and Drug Administration at the time of this study included mainly bisphosphonates, teriparatide, denosumab, raloxifene, and calcitonin [15, 16]. The start time was classified as < 2 years before the initial PFF, between the initial PFF and contralateral PFF, < 1 year after the contralateral PFF, or never (the “never” category included > 2 years before the initial fracture and > 1 year after the contralateral hip fracture). Mortality rates were counted from the contralateral hip fracture to 30 days, 1 year, and 2 years. Among the patients who had never received anti-osteoporosis medications and DXA scanning, we investigated 20 patients or their families and summarized the main reasons for not initiating osteoporosis treatment. A questionnaire survey was conducted to enable all patients who had never received anti-osteoporosis medications and DXA scanning to select the reason.

### Statistical analysis

The concentration and dispersion trends of non-normally distributed data are presented as median and quartile. The paired t-test was used to compare the differences between the two groups of measurement data. The McNemar test was used to compare the difference in the count data of two paired samples. For r × c contingency table data, Chi-square tests were performed to compare the differences between groups. A value of *P* < 0.05 was considered statistically significant. IBM SPSS Statistics 20.0 software was used to perform the statistical analysis.

## Results

### Demographic characteristics and fracture features

In this retrospective study, 181 patients met the inclusion criteria and completed the follow-up. The study population comprised 60 (33.1%) men and 121 (66.9%) women. The median ages of patients with initial PFF and subsequent contralateral PFF were 80 years (range 49–96 years) and 82 years (range 52–96 years), respectively.

**Fig. 1:**
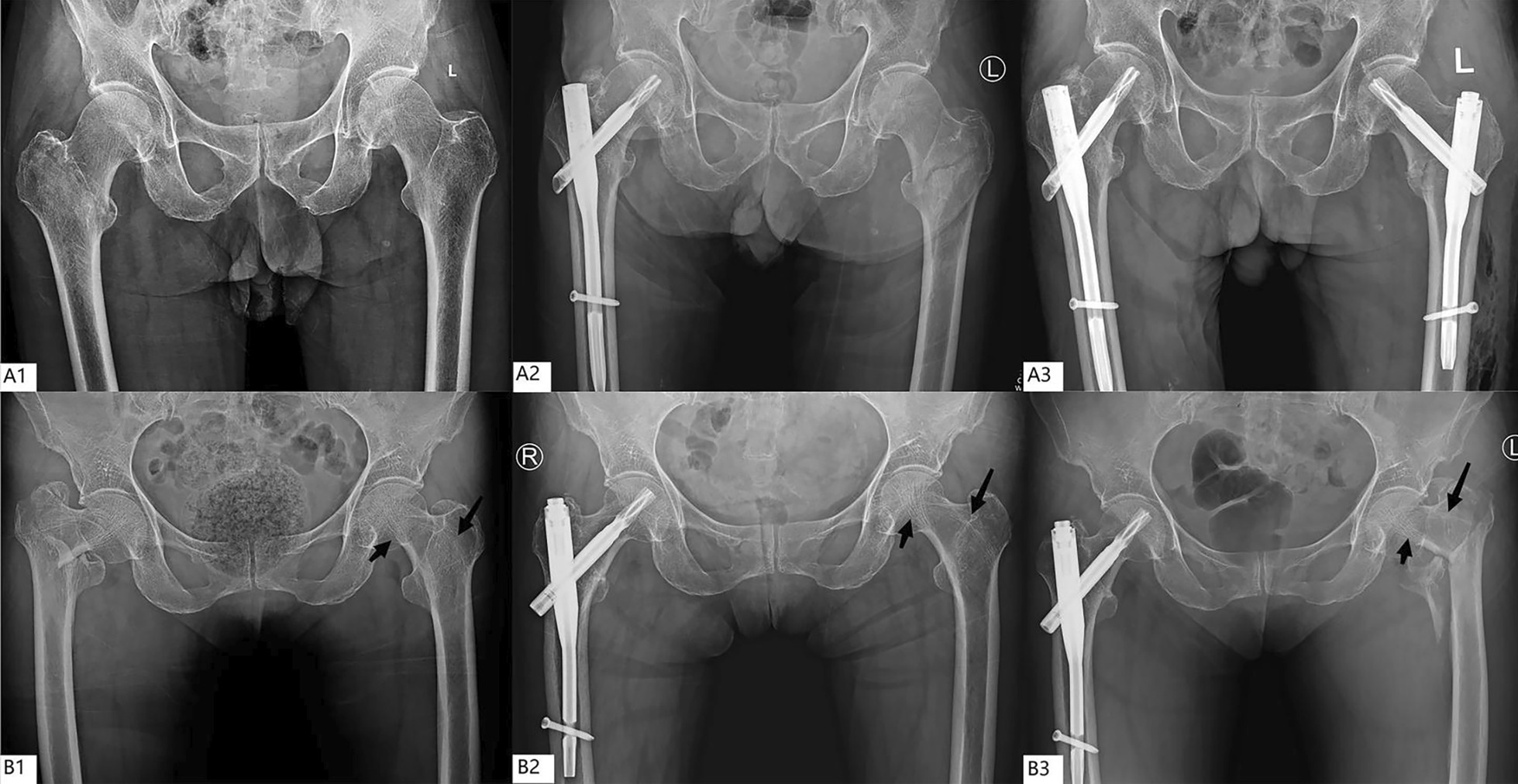
Changes in Singh index in patients with PFF. **A** The Singh index of both proximal femurs was grade I at the time of the initial fracture and remained grade I when the contralateral fracture occurred. **B** Tension trabeculae (short arrows) and pressure trabeculae (long arrows) at the proximal femur were visible at the time of the first fracture, and the tension trabeculae disappeared and pressure trabeculae decreased in severity at the time of the contralateral fracture

The median hospital day of the first fracture was 1 day less than that of the second fracture, and the difference was statistically significant (*P* < 0.05). The injury mechanism of most of the initial and subsequent fractures was falling from the position of standing or sitting height (97.8% and 98.9%, respectively). Almost no high-energy injuries occurred. The Singh index was mostly grade I or II in the contralateral hip at the initial and subsequent fracture, with no statistically significant difference (*P* > 0.05). Most patients already had grade I osteoporosis at the time of the initial fracture, and a small number of patients had mild osteoporosis at the time of the initial fracture; the osteoporosis was further aggravated when the contralateral fracture was detected (Fig. 1). With respect to the surgical procedure for the first fracture, proximal femoral nail antirotation accounted for 33.1%, total hip arthroplasty 10.5%, and hemi-arthroplasty 38.7%. For the second fracture, total hip arthroplasty accounted for 0.0%, proximal femoral nail antirotation 54.1%, and hemi-arthroplasty 40.3% (Table 1). The median interval from the initial fracture to the contralateral fracture was 24 months. The proportion of patients who sustained a subsequent fracture within 3 months to 1 year was the highest at 28.7%. In more than half of the patients (56.3%) with subsequent fractures, the subsequent fracture usually occurred within 2 years of the first fracture. The mortality rates at 30 days, 1 year, and 2 years after surgery for subsequent PFF were 2.2%, 19.9%, and 33.7%, respectively. A total of 130 (71.8%) patients had the same fracture type. Three subtrochanteric fractures were combined with intertrochanteric fractures in the contralateral fracture, and there was no statistically significant difference in the fracture type (*P* > 0.05) (Table 2). Only one patient had an A1.1 fracture, which may not have been classified as an intertrochanteric fracture in the past. Fragility fractures in patients of advanced age are mainly intertrochanteric and femoral neck fractures; subtrochanteric fractures are rare. Therefore, in the present study, we mainly compared the classification of intertrochanteric and femoral neck fractures. There was no significant difference in the classification of stability of femoral neck and intertrochanteric fractures in the comparison between the two groups (*P* > 0.05).

**Table 1:** Univariate analysis between initial and subsequent (contralateral) fracture groups

	First fracture	Contralateral hip fracture	*P* value
Gender			
Male		60 (33.1%)	
Female		121 (66.9%)	
Mean age	80 (75, 84) Range 49–96	82 (77, 88) range 52–96	
Mean hospital day	8 (7, 11)	9 (7, 11)	0.001
Mechanism of injury			
Fall	177 (97.8%)	179 (98.9%)	
Traffic accident	4 (2.2%)	1 (0.6%)	
Fall from high	0	1 (0.6%)	
Singh index			0.213
I	107	118	
II	66	60	
III and above	8	3	
Surgical procedure			0.000
PFNA, Intramedullary nail	67 (37.0%)	100 (55.2%)	
DHS, plate, cannulated screw, FNS	25 (13.8)	8 (4.4%)	
Arthroplasty	89 (49.2)	73 (40.3%)	
Mean Fracture-fracture intervals (months)		24 (7, 36)	
Within 3 months		12 (6.6%)	
3 months–1 year		52 (28.7%)	
1–2 years		38 (21.0%)	
2–5 years		43 (23.8%)	
5 + years		36 (19.9%)	
Mortality			
Within 30 days		4 (2.2%)	
Within 1 year		32 (17.7%)	
Within 2 year		25 (13.8%)	
More than 2 years		120 (66.3%)	

*PFNA* proximal femoral nail antirotation, *DHS* dynamic hip screw, *FNS* femoral neck system

**Table 2:** Comparison of fracture types and fracture classifications between initial and subsequent (contralateral) fracture groups

	Initial fracture	Contralateral hip fracture	*P* value
Fracture type			0.135
Femoral neck	90 (49.7%)	71 (39.2%)	
Intertrochanteric	91 (50.3%)	107 (59.1%)	
Subtrochanteric	0 (0%)	3 (1.7%)	
Fracture classification			
Femoral neck	90 (100.0%)	71 (100.0%)	0.893
Stable patterns	27 (30.0%)	22 (31.0%)	
B1.1	11 (12.2%)	10 (14.1%)	
B1.2	2 (2.2%)	3 (4.2%)	
B2.1	3 (3.3%)	1 (1.4%)	
B3	11 (12.2%)	8 (11.3%)	
Unstable patterns	63 (70.0%)	49 (69.0%)	
B1.3	55 (61.1%)	46 (64.8%)	
B2.2	5 (5.6%)	3 (4.2%)	
B2.3	3 (3.3%)	0 (0%)	
Intertrochanteric	91 (100.0%)	107 (100%)	0.347
Stable patterns	77 (84.6%)	85 (79.4%)	
A1.2	19 (20.9%)	22 (20.6%)	
A1.3	58 (63.7%)	63 (58.9%)	
Unstable patterns	14 (15.4%)	22 (20.6%)	
A1.1	0	1 (0.9%)	
A2.2	3 (3.3%)	8 (7.5%)	
A2.3	5 (5.5%)	6 (5.6%)	
A3.1	2 (2.2%)	2 (1.9%)	
A3.2	1 (1.1%)	1 (0.9%)	
A3.3	3 (3.3%)	4 (3.7%)	

Femoral neck and intertrochanteric fractures were classified by the 2018 AO classification. B1.1, B1.2, B2.1, and B3 represent the stable patterns of femoral neck fractures. A.1.2 and A1.3 represent the stable patterns of intertrochanteric fractures. Subtrochanteric fractures were present in only three patients at the second fracture, and the stability of these fractures is not discussed

### Osteoporosis treatment and examination rates

**Fig. 2:**
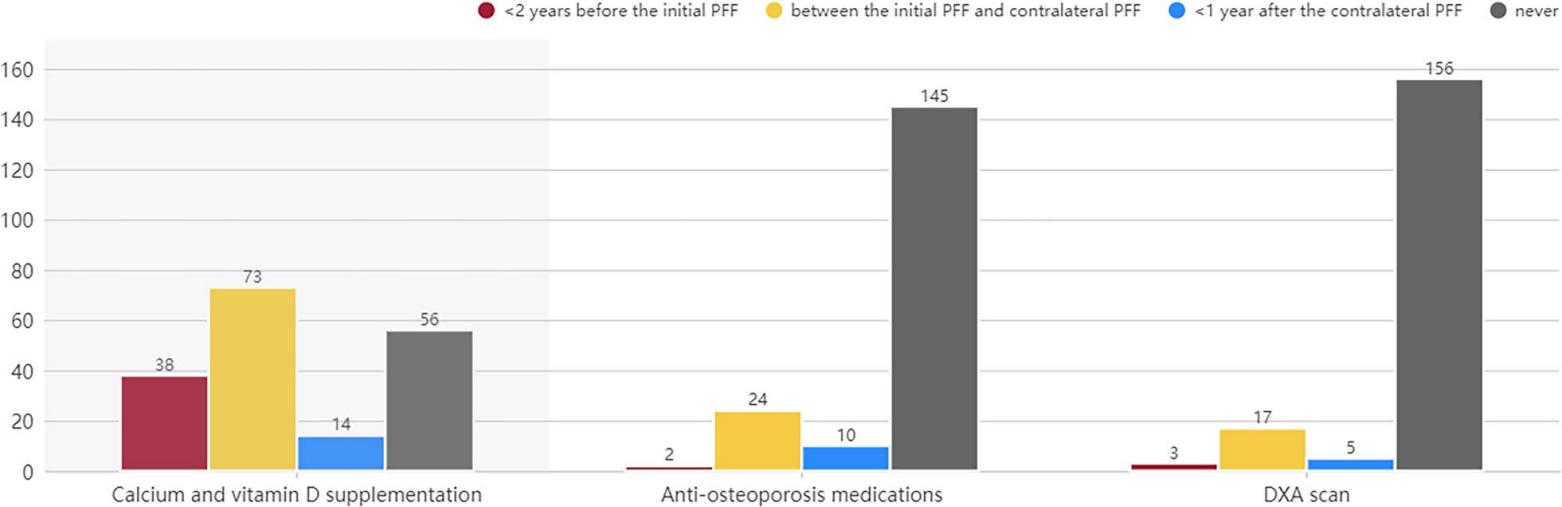
Survey of treatment and examination of osteoporosis in patients with PFF. **A** Calcium and vitamin D supplementation. **B** Anti-osteoporosis medications. **C** DXA scan

Of all 181 patients in this study, 125 (69.1%) received calcium and vitamin D supplementation within the specified time, 24 (13.3%) received anti-osteoporosis medications and DXA scanning, and 144 (79.6%) received neither anti-osteoporosis treatment nor DXA scanning. The numbers of patients who received calcium and vitamin D supplementation < 2 years before the initial PFF, between the initial PFF and the contralateral PFF, and < 1 year after the contralateral PFF were 38 (21.0%), 73 (40.3%), and 14 (7.7%), respectively; the numbers of patients who received anti-osteoporotic medication within these time periods were 2 (1.1%), 24 (13.3%), and 10 (5.5%), respectively; and the numbers of patients who received DXA scans within these time periods were 3 (1.7%), 17 (9.4%), and 5 (2.8%), respectively (Fig. 2).

### Reasons for non-treatment

**Fig. 3:**
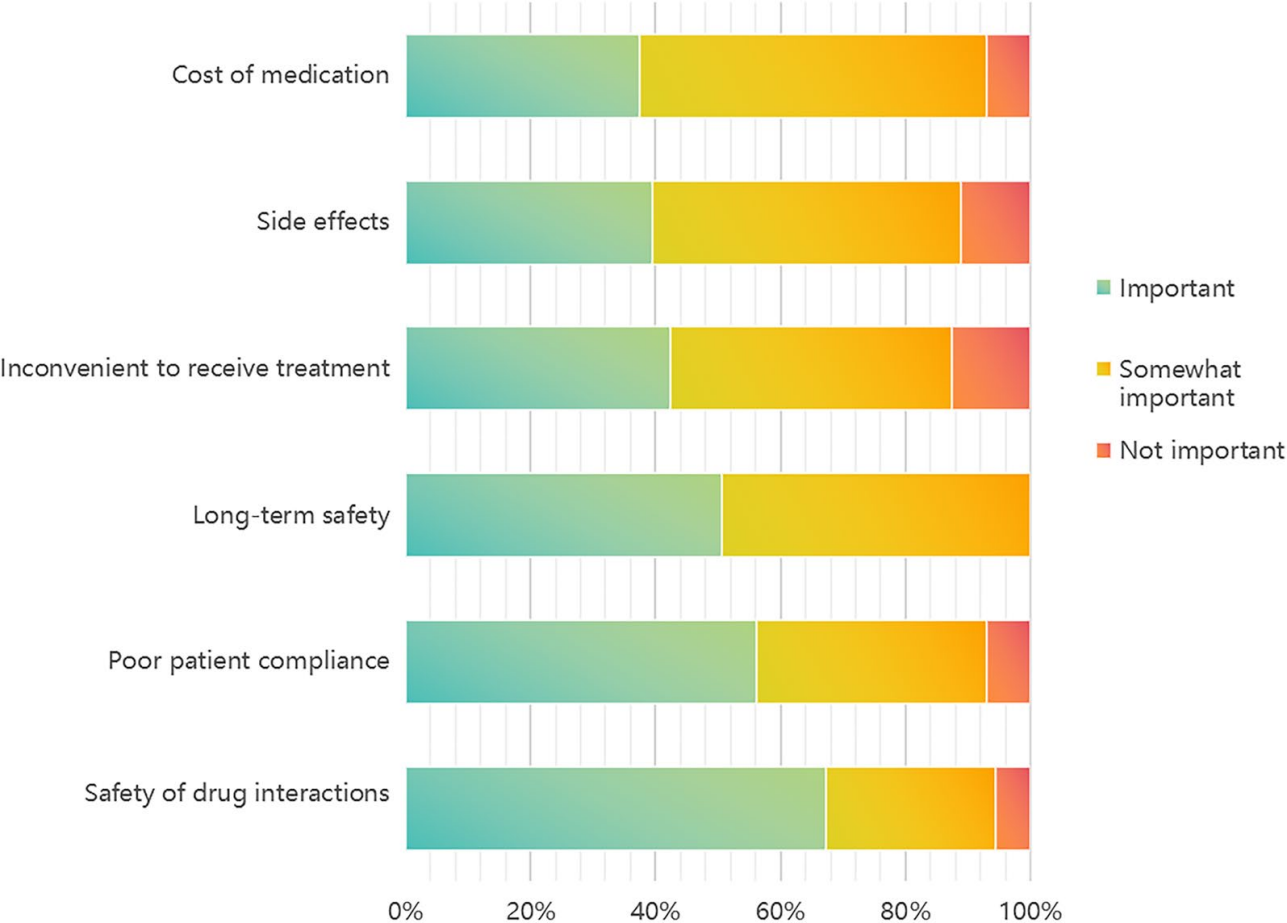
Respondents’ self-reported reasons for not receiving osteoporosis medication

A questionnaire survey was conducted among 144 patients who did not receive anti-osteoporosis medications or DXA scanning. This survey revealed that the main reason for not receiving osteoporosis treatment was concern about the safety of drug interactions (67.4%, important). Other reasons included poor patient compliance, inconvenience of receiving treatment, concern regarding side effects, concern regarding long-term safety, and the costs of the drugs (Fig. 3).

## Discussion

Osteoporosis-related fractures are an important economic burden to medical insurance institutions. Among patients with such fractures, those with hip fracture have the highest proportion of hospitalization and are generally older than patients with vertebral and distal radius fractures. The treatment costs are also much higher than those of other types of fractures [2, 17]. Williams et al. [18] found that during a 12-month follow-up, hip fractures had the highest incremental costs in comparison with the nonfracture cohort. Patients who experienced a second fracture had a higher all-cause health care cost than those who did not sustain a second fracture. In the present study, patients with subsequent PFF were older and had severe osteoporosis, and their Singh index was usually only grade I or II. Although there was no significant difference between the first and second fracture stability patterns of intertrochanteric fracture, in practice it was often more difficult to reset the second intertrochanteric fracture, and there were more fracture complications. All these factors increased the difficulty of patient management. These factors combined with the high number of comorbidities, poor physical condition, and longer perioperative period resulted in a significant difference in the length of stay between the contralateral fracture and the first fracture (*P* < 0.05). A multidisciplinary co-management protocol is often needed for patients with subsequent contralateral PFF. This care protocol combines a variety of experts including emergency specialists, orthopedic surgeons, geriatricians, anesthetists, cardiologists, rehabilitation physicians, neurologists, osteoporosis treatment center staff, post-acute care referral teams, and nursing teams. Li et al. [19] showed that a multidisciplinary co-management protocol for geriatric PFFs can reduce the length of stay, medical expenditures, and mortality during acute admissions.

The patient’s age, skeletal site of initial fracture, history of fracture, osteoporosis, adherence, and weakness strongly influence the magnitude of the subsequent fracture risk [4, 20, 21]. Souder et al. [22] found that patients who underwent closed reduction and percutaneous pinning as the initial treatment for hip fracture due to a shortened leg length had a higher risk of subsequent contralateral hip fracture than patients who underwent arthroplasty. The mechanical stability of modified femoral salvage internal fixation has improved in recent years, and the risk of re-fracture may be reduced [23]. There was a statistically significant difference between the two surgical procedures in this study; the increase in internal fixation was associated with a higher incidence of intertrochanteric fracture among the second fractures. Most of the contralateral PFFs were of the same type as the initial fracture, suggesting that that specific hip morphology and gait factors are likely to lead to different fracture types [24, 25]. The median fracture interval was 24 months. More than half of the patients had subsequent contralateral PFFs within 2 years of the initial fractures, and the possibility of subsequent fracture in the first year was the highest. These findings are similar to the results of previous studies [23, 26].

Galler et al. [27] conducted a telephone follow-up of patients with PFF who underwent surgery from 2006 to 2007, and more than half of all patients had already died 5 years after the initial surgery. This mortality rate is appalling in patients with fracture. In an epidemiological study of contralateral PFF conducted by Murena et al. [26], patients with contralateral fracture had a significantly lower body mass index and a significantly lower proportion of malnourishment, and the 1-year mortality rate of the contralateral PFF was 20.5% (significantly lower than that of the first PFF). This proportion is similar to that in our study (19.9%). One reason for this result might be the higher mortality rate after the first fracture in patients with severe comorbid conditions. Some authors have also reported lower mortality in association with subsequent fractures [28].

The Singh index can indicate whether patients need DXA scanning and anti-osteoporosis treatment. One study showed that the Singh index of the unfractured hip at the time of the first hip fracture was significantly lower at the time of the second hip fracture [29]. Most patients in the present study had severe osteoporosis at the time of the first fracture as measured by the Singh index. The Singh index on the side of the fracture at the time of the second contralateral PFF was not significantly different from the Singh index at the time of the first fracture (*P* > 0.05). Almost all patients had a fracture caused by low-energy injury, which was closely associated with severe osteoporosis. Treatment of osteoporosis should be undertaken early to improve bone density and prevent hip fractures [30, 31].

Many studies have shown that there is a gap between osteoporosis diagnosis and treatment after a fragility fracture [7, 16, 32]. Implementation of DXA screening and anti-osteoporosis medications is severely inadequate. Ramachandran et al. [33] found that patients aged > 75 years or with a low income subsidiary status had lower rates of osteoporosis examination and treatment. In addition, patient awareness of osteoporosis is related to the disease type and fracture site. Patients with PFF are generally less aware of osteoporosis than osteoarthritis, and patients with distal femoral fracture are generally less aware of osteoporosis than PFF [34, 35]. Patients with fractures are often not informed of the risks of not receiving osteoporosis treatment. Patients with distal femoral fractures have lower limitations in their quality of life, do not have repeated outpatient reviews and so are rarely informed of the risk of osteoporosis. Making patients aware of the risk of osteoporosis should also be an important part of the treatment of patients with fractures. The lower hazard of all-cause mortality was significant in patients treated for osteoporosis after sustaining a fracture. In the present study, 66.7% of patients receiving anti-osteoporosis medications began treatment after the initial fracture, and only 5.6% of the osteoporosis treatments occurred before the initial fracture. BMD assessment significantly increases the initiation of osteoporosis treatment [36]. BMD assessment provides the basis for the prescription of anti-osteoporosis medications. Additionally, orthopedic surgeons can more reasonably use anti-osteoporosis medications based on BMD assessment, and patients are also more receptive. We may consider adding BMD assessment to our routine admission examination of advanced-age patients. Barton et al. [37] conducted two digital surveys of all orthopedic surgeons and dedicated orthopedic midlevel clinicians at a level 1 trauma center in the United States. They believed that orthopedic surgeons are well situated to address this care gap of osteoporosis. Most respondents perceived osteoporosis care to be very important. However, the respondents were more willing to use calcium and vitamin D than anti-osteoporosis medications. The greatest reason for not prescribing anti-osteoporosis therapy was limited experience with that therapy. Orthopedic surgeons and patients had misperceptions about the side effects of anti-osteoporosis medications. Although side effects of osteoporosis medications exist, they are rare at the currently recommended doses [38–40].

Lindsay et al. [40] investigated the reasons why postmenopausal women diagnosed with osteoporosis do not receive osteoporosis treatment in the United States. The most important reasons were the use of alternative therapies such as over-the-counter vitamins/supplements and the fear of side effects. Yu et al. [41] found that the main concern among women with osteoporosis who do not receive anti-osteoporosis medications is side effects, and the drug cost was the reason why 34.1% of the women did not start treatment. In our study, concern about the safety of drug interactions was the main reason for lack of osteoporosis treatment in patients with subsequent contralateral PFF. The patients were of advanced age and were already taking many drugs to treat basic diseases. Studies have shown that most drugs used to treat osteoporosis are safe for patients with cardiologic disease [42, 43], revealing no significant difference in the total incidence of atrial fibrillation and cardiovascular adverse events between bisphosphonates and placebo. Cardiology drugs such as statins, beta-adrenergic antagonists, and thiazides may provide additional benefit to the treatment of osteoporosis. Another review showed that people who take calcium supplements for a long time have a significantly increased risk of myocardial infarction and coronary artery calcification [44]. The best management method for advanced-age patients with osteoporosis and multiple diseases has not yet been determined. Guidelines for the treatment of osteoporosis in older adults with different comorbidities need to be proposed. We should strengthen the science popularization of osteoporosis treatment at both the patient level and doctor level to reduce the risk of fracture. We also need improvements in the nursing system to facilitate the treatment of osteoporosis for patients who have fracture with poor mobility.

The main limitation of this study is that patients with a long fracture interval may have been excluded because of incomplete data, resulting in bias. We did not compare the data of patients with secondary fragile fractures in other parts of the body, such as proximal humerus fractures, distal radius fractures, and vertebral body fractures. Telephone surveys are limited by the accuracy of patient recall and reporting bias. In this study, osteoporosis treatment and DXA scanning were based on self-reports and were not confirmed through medical records or contact with doctors; therefore, errors were prone to occur. When the patients were asked if they had ever had a DXA scan, recall bias and lack of familiarity with terminology may have affected their response to this question.

## Conclusions

This study has shown that patients with subsequent contralateral PFF are mostly of advanced age, have a higher proportion of intertrochanteric femoral fractures, have more severe osteoporosis, and have longer hospital stays. The difficulty in managing such patients requires multidisciplinary involvement. These patients often are not evaluated or do not receive anti-osteoporosis treatment. Anti-osteoporosis medications and examinations need to be popularized. Advanced-age patients with osteoporosis need more reasonable treatment and management methods.

## Data Availability

Not applicable.

## Abbreviations

PFF: Proximal femoral fracture
BMD: Bone mineral density
DXA: Dual X-ray absorptiometry
