# Correction: Characteristics of subsequent contralateral proximal femoral fracture: more convenient access needed to treat osteoporosis

**DOI:** 10.1186/s13018-023-03741-5

**Published:** 2023-04-19

**Authors:** Yuxuan Jiang, Yangjun Zhu, Binfei Zhang, Dongxu Feng

**Affiliations:** 1 Department of Orthopaedic Trauma, Hong Hui Hospital, Xi’an Jiaotong University School of Medicine, Xi’an, 710054 Shaanxi Province China; 2 Department of Joint Surgery, Hong Hui Hospital, Xi’an Jiaotong University School of Medicine, Xi’an, 710054 Shaanxi Province China

**Correction: Journal of Orthopaedic Surgery and Research (2023) 18:126** 10.1186/s13018-023-03621-y

Following publication of the original article [1] the authors have identified an error in correspondence. The correct version of corresponding author is shown below.

The authors apologize for the error.

The original article has been corrected.
